# Woman with a Painful Rash

**DOI:** 10.5811/cpcem.20735

**Published:** 2025-01-12

**Authors:** Leyan Shalabi, Wesley Eilbert

**Affiliations:** University of Illinois at Chicago, College of Medicine, Department of Emergency Medicine, Chicago, Illinois

**Keywords:** eczema herpeticum, eczema, atopic dermatitis, herpes simplex virus

## Abstract

**Case presentation:**

A 21-year-old woman with a history of eczema presented to the emergency department with a painful rash over the previous three days spreading from her left axilla to her left arm, left chest and left abdominal wall. The rash consisted of clusters of small, erythematous vesicles on hyperpigmented patches of skin. The patient was treated empirically with intravenous acyclovir for eczema herpeticum with improvement. Polymerase chain reaction testing of the fluid obtained from the rash vesicles later confirmed the presence of herpes simplex virus-1.

**Discussion:**

Eczema herpeticum is a cutaneous superinfection with herpes simplex virus on pre-existing sites of eczema. Left untreated, it can have a mortality rate over 50%. Early identification and treatment of this high morbidity condition with antiviral agents is key to improving outcome.

## CASE PRESENTATION

**Image 1:**
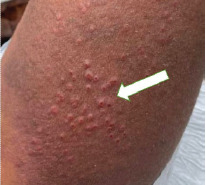
Erythematous vesicles clustered on a hyperpigmented patch on the patient’s left upper arm (arrow).

**Image 2:**
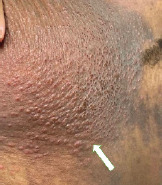
Erythematous vesicles clustered on a hyperpigmented patch on the patient’s left chest wall (arrow).

**Image 3:**
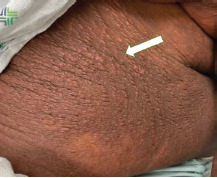
Erythematous vesicles clustered on a hyperpigmented patch on the patient’s left abdominal wall (arrow).

A 21-year-old woman with a history of eczema presented to the emergency department complaining of a painful rash present for three days. Over the previous week, she had been experiencing worsening eczema symptoms for which she had been applying two corticosteroid creams. The painful rash consisted of grouped clusters of small erythematous vesicles overlying hyperpigmented patches. The rash had started in her left axilla and spread to her left arm, chest, and abdomen (Images 1, 2 and 3). With the rash, she reported associated chills and intermittent emesis.

Due to the extensive skin involvement and associated systemic symptoms, the patient was treated empirically with intravenous acyclovir and admitted to the hospital. Fluid collected from the vesicles of the rash was positive for herpes simplex virus (HSV) -1 deoxyribonucleic acid (DNA) on polymerase chain reaction (PCR) testing. She had significant improvement of the rash and was discharged on hospital day three to complete a 10-day course of valacyclovir.

## DISCUSSION

Atopic dermatitis, or eczema, is the most common inflammatory skin disease, affecting up to 18% of children and 7% of adults.^1^ Eczema herpeticum (EH) is a cutaneous superinfection with HSV, most commonly HSV-1, on pre-existing sites of eczema.^2^,^3^ Up to 3% of patients with eczema will experience an episode of EH, and at least 20% of all patients with EH will have a history of recurrent herpes infections.^1^,^4^ An initially local disease, EH may progress to a potentially life-threatening systemic infection.^3^

Eczema herpeticum typically presents as a sudden eruption of monomorphic, dome-shaped, grouped, 2–3 millimeter vesicles on an erythematous base, superimposed on areas of pre-existing sites of eczema, most commonly the face, neck and upper chest.^5^ The rash is pruritic and painful and may spread to involve areas of normal skin.^5^ The rash is often accompanied by systemic symptoms such as fever, malaise, headache, and lymphadenopathy.^4^ The vesicles rupture and form crusts over underlying erosions after one to two weeks.^3^

The diagnosis of EH is made on clinical grounds and confirmed by the detection of HSV DNA in vesicle fluid by PCR.^5^ The sensitivity of PCR testing is between 80–100%.^3^ If PCR is not available, direct fluorescent antibody testing, a Tzank smear, or viral cultures may be used.^5^

The mortality rate of EH in the era before antiviral therapy was frequently over 50%.^6^ Given its potential high morbidity, treatment of EH should begin when it is suspected, without waiting for confirmatory tests.^1^,^3^ Acyclovir is the antiviral agent of choice for the treatment of EH.^3^,^4^ Mild cases can be treated with oral acyclovir on an outpatient basis.^5^ Patients with signs of systemic illness, extensive skin involvement, and those less than one year of age should be hospitalized and treated with IV acyclovir.^1^,^5^ Up to 30% of patients hospitalized for EH will have bacterial superinfection with *Staphylococcus aureus*, and some authors recommend empiric treatment with antistaphylococcal antibiotics for cases with extensive skin involvement.^7^,^8^

**CPC-EM Capsule**

What do we already know about this clinical entity?

*Eczema herpeticum is a cutaneous superinfection with herpes simplex virus. Left untreated, eczema herpeticum can have a mortality rate over 50%*.

What is the major impact of the image(s)?

*The images show the characteristic rash of eczema herpeticum: grouped clusters of small erythematous vesicles overlying hyperpigmented patches of skin*.

How might this improve emergency medicine practice?

*Rapid identification and treatment is necessary to prevent associated mortality*.
